# Editorial: Trends in digital hearing health and computational audiology

**DOI:** 10.3389/fnins.2024.1522600

**Published:** 2024-11-26

**Authors:** Faheema Mahomed-Asmail, Karina De Sousa, Laura Coco

**Affiliations:** ^1^Department of Speech-Language Pathology and Audiology, University of Pretoria, Pretoria, South Africa; ^2^School of Speech, Language and Hearing Sciences, San Diego State University, San Diego, CA, United States

**Keywords:** digital hearing health, computational audiology, telehealth, AI in hearing care, e-audiology, digital transformation

## Introduction

Traditional hearing health care (HHC) service delivery models focus on face-to-face, clinic-based testing, often requiring several patient visits (World Health Organization, 2013). However, access to these services remains limited globally, leaving millions with untreated hearing loss, which has pervasive and profound consequences (Olusanya et al., 2014; Shukla et al., 2020). The shift toward mHealth and modern machine learning present opportunities to increase access in HHC through scalable models of care. This can be facilitated by low-cost hearing devices, smartphone technologies, and equipping a larger number of specialists for medical and surgical management of ear and hearing diseases (Bernstein et al., 2018). Furthermore, computational auditory models, advanced algorithms, and the use of artificial intelligence offer promising avenues for developing new hearing solutions and optimizing existing ones (Boisvert et al., 2023).

This Research Topic aimed to collect the latest research in these areas to support the effective implementation of digital technologies and computational methods in order to improve accessibility to ear and hearing healthcare services. The special edition consists of 11 articles and spanned over two Frontiers journals, Frontiers in Neuroscience, and Frontiers in Audiology and Otology. The Research Topic was initiated in June 2023, and opened for submission from September 2023 to October 2024, with a total of 14 submissions being received. The papers included in this edition are broad in their scope, ranging from validation of automated audiometry to machine learning and artificial intelligence.

## Advances in audiometric assessment and hearing conservation methods

Automated audiometry has been proposed as an alternative of diagnostic assessment to improve access to hearing care by reducing time and costs, especially in areas with limited specialist availability. Liu et al. examined the correlation of air-conduction thresholds between automated audiometry conducted in a non-isolated environment and manual audiometry performed in a soundproof setting on individuals with normal hearing and varying degrees of hearing loss. Consistent with previous research (Corry et al., 2017; Mahomed et al., 2013), Liu et al. found comparable results between the two methods across hearing levels.

Hearing conservation programs rely on serial audiograms to monitor shifts in hearing over time. McMillan et al. identified limitations in traditional approaches to serial monitoring and proposed a new statistical modeling method using a Gaussian process. This approach enables individualized predictions and simplifies interpretation, providing a less biased, more accessible tool for early detection of hearing changes.

## Speech-in-noise testing

Human communication often occurs under adverse acoustical conditions, where speech signals mix with interfering speech or noise. Speech-in-noise (SIN) audiometry is thus a valuable part of audiological diagnostics and clinical measurements. Génin et al. developed and normalized a French speech-in-noise (SIN) test, SoNoise, to use as both a screening and a clinical evaluation tool. Normative values for diotic and antiphasic presentations were established with findings accurately capturing SIN abilities across various populations. Whereas, Meyer et al. investigated the use of a humanoid NAO robot to present target sentences alongside competing masker speech in a speech-in-speech test framework. Functional similarity was found in speech intelligibility when the NAO robot was compared to a traditional computer setup, with participants generally positive toward robot interactions.

## Hearing aid technology and user experience

Hearing aids (HA) are prescribed to enhance communication and improve the quality of life for those who have hearing loss, but many individuals do not wear them consistently due to discomfort, dissatisfaction, or perceived lack of benefit, especially in noisy environments (Heselton et al., 2022). Alishbayli et al. developed a fast, domain-free noise suppression method, Statistical Sound Filtering (SSF), which used sound textures' statistical properties to enhance speech clarity. The evaluation of SSF demonstrated improvements in sound quality and reduced background noise levels without compromising speech intelligibility suggesting that SSF could be effectively integrated into HAs. While Fourie et al. examined the positive experiences of HA users through ecological momentary assessment (EMA) and found significant benefits in various contexts, particularly in conversational settings and leisure activities. Similarly, Sheng et al. investigated the perceived benefits of over-the-counter (OTC) hearing aids, a recently launched category of hearing devices, revealing that users experienced satisfaction scores comparable to traditional hearing aid users, along with notable improvements in emotional health, relationships, and communication abilities.

Linked to intervention options, Madahana et al. developed and tested a monitoring system that integrates a smartwatch and smart hearing muff with sound sensors in a mock mine environment. The system effectively detected noise levels and successfully communicated alerts to miners; however, further refinements and testing are required. Similarly, Andersson et al. leveraged EMA on heart rate data to understand the factors influencing real-world listening experiences. Results from a preliminary study among individuals with no hearing loss indicated that momentary heart rate data helped improve the prediction of self-reported listening experiences (passive vs. active listening). This study underscores the potential of integrating physiologic EMA data to deepen our understanding of listening dynamics in everyday environments and suggests promising applications for improving hearing aid outcomes among individuals with hearing loss.

## Auditory training and assessment for pediatric populations

Spatial hearing is crucial for communicating in noise and can improve with training. Parmar et al. present a novel virtual reality (VR) game for an intervention designed to enhance spatial hearing in children and young people with bilateral cochlear implants. The BEARS (Both Ears) approach leverages the engaging, interactive, and immersive format of VR to strengthen listening skills, with the aim of supporting communication skills in noisy environments.

Auditory processing disorder (APD) assessments present challenges due to the disorder's heterogeneous nature, necessitating significant experience and training for accurate diagnosis. Wimalarathna et al. used a Random Forest model to analyse data from APD clinical test batteries to categorize children with APD into specific clinical subgroups which achieved 90% accuracy.

## Conclusion

This Research Topic of articles highlights innovative solutions that can significantly enhance the accessibility and effectiveness of ear and hearing healthcare services, addressing the critical need for more inclusive approaches to managing hearing health across diverse populations.
